# Landscape structure affects distribution of potential disease vectors (Diptera: Culicidae)

**DOI:** 10.1186/s13071-017-2140-6

**Published:** 2017-04-26

**Authors:** Carina Zittra, Simon Vitecek, Adelheid G. Obwaller, Heidemarie Rossiter, Barbara Eigner, Thomas Zechmeister, Johann Waringer, Hans-Peter Fuehrer

**Affiliations:** 1 Institute of Parasitology, Department of Pathobiology, University of Veterinary Medicine, Vienna, Austria; 2 Department of Limnology and Bio-Oceanography, University of Vienna, Vienna, Austria; 3 Federal Ministry of Defence and Sports, Division of Science, Research and Development, Vienna, Austria; 4 Donaustraße 73, 3421 Höflein/Donau, Austria; 5 Biological Station Lake Neusiedl, Burgenland, Austria

**Keywords:** Culicidae, Mosquito communities, Canonical correspondence analysis, Autecology, Species distribution patterns, Seasonality, Non-metric multidimensional scaling

## Abstract

**Background:**

Vector-pathogen dynamics are controlled by fluctuations of potential vector communities, such as the Culicidae. Assessment of mosquito community diversity and, in particular, identification of environmental parameters shaping these communities is therefore of key importance for the design of adequate surveillance approaches. In this study, we assess effects of climatic parameters and habitat structure on mosquito communities in eastern Austria to deliver these highly relevant baseline data.

**Methods:**

Female mosquitoes were sampled twice a month from April to October 2014 and 2015 at 35 permanent and 23 non-permanent trapping sites using carbon dioxide-baited traps. Differences in spatial and seasonal abundance patterns of Culicidae taxa were identified using likelihood ratio tests; possible effects of environmental parameters on seasonal and spatial mosquito distribution were analysed using multivariate statistical methods. We assessed community responses to environmental parameters based on 14-day-average values that affect ontogenesis.

**Results:**

Altogether 29,734 female mosquitoes were collected, and 21 of 42 native as well as two of four non-native mosquito species were reconfirmed in eastern Austria. Statistical analyses revealed significant differences in mosquito abundance between sampling years and provinces. Incidence and abundance patterns were found to be linked to 14-day mean sunshine duration, humidity, water–level maxima and the amount of precipitation. However, land cover classes were found to be the most important factor, effectively assigning both indigenous and non-native mosquito species to various communities, which responded differentially to environmental variables.

**Conclusions:**

These findings thus underline the significance of non-climatic variables for future mosquito prediction models and the necessity to consider these in mosquito surveillance programmes.

**Electronic supplementary material:**

The online version of this article (doi:10.1186/s13071-017-2140-6) contains supplementary material, which is available to authorized users.

## Background

Distribution, endemicity and transmission potential of vector-borne pathogens is regulated by the communities of potential vector organisms [1, 2]. Mosquitoes, while mostly a nuisance of little impact, are also among the most important vectors for various pathogens. Several Culicidae taxa have been demonstrated to (potentially) transmit members of the *Flaviviridae* (e.g. West Nile virus, Japanese encephalitis virus, dengue virus, Usutu virus) and the *Togaviridae* (e.g. sindbis virus), and various endo-parasites like *Plasmodium* spp. and *Dirofilaria* spp. [3–5]. However, each mosquito-borne pathogen can only be successfully transmitted by a specific range of suitable mosquito species. Therefore, a better knowledge of the species-specific responses to environmental parameters influencing spatial and temporal mosquito species distribution variation, seasonality and community composition is crucial to the understanding and prediction of vector-pathogen dynamics. In particular, spatiotemporal occurrence and abundance patterns of potential vector species are essential in defining where and when vector-borne pathogens might spread [6]. Moreover, environmental conditions must allow the completion of the life-cycle of the pathogens’ primary and secondary hosts [7, 8]. Epidemics involving humans or domesticated animals may only emerge if pathogens, vectors, and potential hosts co-occur [8], and vectors target potential hosts indiscriminately.

Mosquito species-specific host-feeding preferences are assumed to influence the vector capacities substantially [9] although pathogen transmission is more likely influenced by host availability instead of species-specific host preferences of potential vectors [10].

As mosquitoes are obligate semi-aquatic insects, mosquito presence and propagation in any given area is controlled by the availability of suitable larval habitats. Adult female mosquitoes will select adequate breeding sites such as ponds, floodplains, or phytothelmata to deposit their eggs [11]. Also, species-specific preferences for certain characteristics of larval habitats have been identified [12, 13]. Consequently, adult mosquitoes are often found in abundance close to their original larval habitats, presence and abundance of which depends on landscape cover, climate and seasonality [14–16]. Landscape elements not only control breeding habitat availability but also govern occurrence and abundance of suitable hosts groups, such as birds [17], amphibians [18] and reptiles [19, 20]. Indeed, effects of landscape factors on mosquito diversity and abundance are increasingly assessed in ecological analyses of mosquito communities [21–23].

Spatial adult mosquito distribution is also linked to host availability, potentially leading to an aggregation of mosquitoes in areas with high densities of hosts and suitable breeding habitats, since successful blood feeding is crucial for the completion of the mosquito life-cycle [19]. Seasonal shifts in host use of mosquito taxa have been found in response to host reproductive phenology, where mosquitoes exploit the most abundant resource as and when it becomes available [24]. Indeed, host preference of different mosquito species was found to strongly overlap resulting in shared host use across several taxa [25]. Supposed aggregation of mosquitoes around human populations is thus likely more decisively controlled by the availability of breeding habitats, as human settlements were mostly established at rivers and close to wetlands [11].

Spatiotemporal patterns of mosquito communities are strongly linked to environmental conditions [26, 27]. Variation of precipitation, accumulated snow pack, and temperature has been shown to affect abundance patterns in various mosquito species [8, 28, 29]. Drought events were found to increase the abundance of wetland mosquitoes, potentially through the exclusion of predators and competitors [30]. Also, ecological niche modelling mostly draws on climatic data [31, 32].

Moreover, and in addition to affecting vector populations, climatic parameters control transmission efficacy of various mosquito-borne pathogens. For instance, incubation temperature controls the ability of *Culex pipiens* L. to transmit West Nile Virus (WNV) [33]. West Nile virus infection rate in *Culex* mosquitoes was also found linked to prior weather conditions [34]. Malaria risk was found to be dependent not only on temperature, affecting mosquito and *Plasmodium* development, but also on precipitation and water quality [35]. Thus, climate change will likely alter the potential propagation of vector-borne diseases [5].

Recently, WNV was identified in blood donors and mosquitoes in Austria [36–38]. In addition, several other mosquito-borne viruses were reported from Austria or neighbouring countries including sindbis virus, Usutu virus, chikungunya virus, Batai virus and Tahyna virus [4, 39–41]. Also, several *Plasmodium* species were discovered in abundance in Austrian birds [42], and human-pathogenic *Plasmodium* species are frequently imported [43]. Filarioid helminths, known to be of veterinary significance and to potentially infest humans, were increasingly being reported from Austria and neighbouring countries [44, 45].

Since potential vectors for these pathogens have been reported from Austria [3], analysis of seasonal and spatial patterns of medically important mosquito species is crucial to be able to estimate the potential impact of vector-borne pathogens.

In this study, we identify mosquito communities present in eastern Austria and analyse spatiotemporal community dynamics in relation to abiotic parameters and habitat structure. In particular, we assess how landscape elements and climatic parameters affect mosquito communities and identify major drivers of mosquito diversity and abundance in eastern Austria. More specifically, we expect the occurrence of single species to be linked to certain types of land cover, assuming that abundances are more distinctly controlled by climatic parameters. We hypothesise that certain species will predominately or exclusively occur in certain types of landscape coverage while exhibiting strong association of phenology and climatic conditions. We predict differentiation of mosquito taxa along landscape gradients and distinct mosquito habitat preferences in response to land cover types. To our knowledge, this study is the first to directly exploit the fine resolution of the CORINE (co-ordinated information on the environment) Land Cover (CLC) database [46] to identify parameters shaping mosquito communities. Thus, we deliver highly relevant background data on vector communities in eastern Austria that are necessary for the optimisation of future mosquito, and mosquito-borne pathogen surveillance approaches.

## Methods

### Mosquito sampling and identification

Within this study, 35 permanent and 23 non-permanent mosquito trapping sites were selected across three provinces of eastern Austria (Burgenland, Lower Austria and Vienna). Female mosquitoes were sampled twice a month, every second week of the month for a 24-h period from April to October 2014 and 2015 using carbon dioxide-baited traps (Biogents Sentinel Traps, Regensburg, Germany). Additionally, the BG-Sentinel traps were operated at least once and up to six times in non-permanent sampling sites during the summer months.

Collected female mosquitoes were stored at -80 °C and morphologically identified using the keys of Becker et al. [11] and Mohrig [47]. Some specimens lacked morphological identification characters and were only identified to genus-level. Genomic DNA from three legs of up to four individuals of each sampled taxon was extracted singly using the DNeasy Tissue Kit according to the manufacturer’s protocol (Quiagen®, Hilden, Germany). Partial sequence amplification of the mitochondrial cytochrome *c* oxidase subunit 1 (*cox*1) gene was performed as reported previously, targeting fragments of approximately 700 bp length [48, 49]: briefly, whole genomic DNA was extracted from legs or the head capsule of single specimens using the DNeasy Blood & Tissue Kit (Qiagen), according to the manufacturer’s protocol. Amplification of a ~ 700 bp-long *cox*1 fragment was achieved using primers H15Culi-COIFw and H15CuliCOIRv in standard PCR protocols [49]. Subsequently, PCR fragments were purified and sequenced by a commercial company (LGC Genomics GmbH, Berlin, Germany) to confirm the morphological identification. Members of the *Cx. pipiens* and the *Anopheles maculipennis* complexes, as well as *Aedes cinereus*/*geminus* were not identified to biotype or species-level as differentiability is not given by the usage of *cox*1. For a detailed analysis of responses of members of the *Cx. pipiens* complex and *Cx. torrentium* to environmental conditions, please consult Zittra et al. [49].

### Statistical analysis

**Fig. 1:**
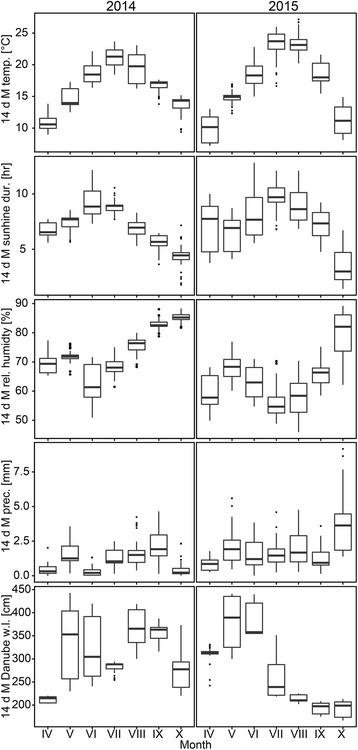
Seasonal variation of environmental parameters within sampling years. From top to bottom, boxplots display observed variation in 14-day mean air temperatures, sunshine duration, relative humidity, precipitation, and average maximum Danube water levels; left panels depict variation of environmental conditions observed in 2014, right panels display patterns observed in 2015

For comparative statistical analysis, only mosquitoes sampled during both sampling seasons by using BG-Sentinel traps were used. Non-permanent sampling sites were not included in the statistical analysis. Proportional differences in abundance of the most abundant mosquito taxa in eastern provinces of Austria (corrected against sampling events and sampling sites per province) collected in 2014 and 2015 were assessed using the William's correction to the G likelihood ratio test (G-test) of goodness-of-fit [50]. We used canonical correspondence analysis (CCA; [51, 52]) to relate mosquito community fluctuation patterns based on abundance responses of single species to environmental parameters. Canonical correspondence analysis is a multivariate direct gradient analysis, known to give a broad overview of multiple taxa and communities [53], and was used to relate taxa to a specific set of environmental variables. High resolution and high-quality land use type as available in the CORINE Land Cover (CLC) database [46] was included as a predictor variable. These data were compiled by the European Community (EC) and were selected due to the high spatial resolution (1:100,000) and the homogeneity of the methodology used for the classification of land cover types. CLC level 3 was chosen because it provides the highest resolution on habitat information within a land cover type. Furthermore, the analysis included scaled weather data (raw data provided by the Austrian official weather service, ZAMG), and Danube water levels (raw data provided by the viadonau GmbH). We used ontogenetically relevant 14-day-mean values prior to each single sampling date to assess effects of meteorological data (relative air humidity, sunshine duration, the amount of precipitation, air temperature and atmospheric pressure) as well as Danube water levels on mosquito communities. Additionally, these data exhibit distinct temporal patterns (Fig. 1), allowing the linkage of relationships established by CCA to the seasonality of mosquito species. Species represented by less than 30 specimens in the whole dataset were excluded from the analysis. Raw species count data of single sampling events were used to build CCA models; for full models, environmental parameters were supplied as 14-day-means only (as 14-day-maxima, 14-day-minima and 14-day-standard deviation were found to exhibit significant collinear trends to 14-day-means) prior to sampling, habitat classes and provinces were provided as dummy-coded categorical variables. Full models including air temperature, precipitation, sunshine duration, distance to the nearest wetland (T. Zuna-Kratky, unpublished), province, CLC level 3 land cover classes of sampling sites, and mean maximum Danube water level were constructed and used to infer most parsimonious models via random addition/removal of model terms. Only results of most parsimonious CCA models are presented. As sites were repeatedly sampled, we tested stratified CCA models assuming random effects of sampling sites to account for pseudo-replication.

Complementarily, effects of environmental parameters influencing Culicidae community composition were assessed using permutational multivariate analysis of variance (PERMANOVA) on dissimilarity matrices [54–56]. We used unique sampling events of communities to construct a dissimilarity matrix and performed PERMANOVA as in [50]. Only sampling events comprising more than ten specimens were used for this analysis. Again, pseudo-replication by multiple sampling of sampling sites was accounted for by including an appropriate error term. Environmental parameters with a significant contribution to the observed patterns were modelled in ordination space to estimate non-linear associations using the function ‘vegan:ordisurf()’.

All statistical analyses and figures were done in the R statistical environment (R Development Core Team, 2011) using ‘*vegan*’ [54], ‘*MASS*’ [57], ‘*shape*’ [58] and ‘*ggplot2*’ [59] packages.

## Results

### Variation of climatic parameters

While not subjected to statistical testing, climatic parameters exhibited distinct patterns between and within years as typical for the region (Fig. 1). Temperature and sunshine duration exhibited unimodal trends and peak in the summer months in both years. Relative humidity, precipitation, and Danube water levels behaved more erratically. Slightly higher humidity was observed in 2014, while precipitation was higher in 2015. Two flood events were observed in 2014, one in early summer and one in late summer/fall, while only one early summer flood event was observed in 2015.

### Species inventory

In total, 29,736 mosquitoes were sampled in 2014 and 2015. Within the two sampling years 21 mosquito species and three mosquito taxa comprising a total of eight species/biotypes were collected in eastern Austria (Table 1). Molecular identification (GenBank® accession numbers KM243936–KM243960, KM280573–KM280597, KU756484–KU756487; [49, 50]) of taxa confirmed standard morphological identification of specimens, indicating a high quality and consistency of taxonomic work. Overall, the most abundant species was *Coquillettidia richiardii* with 31.36% (*n* = 9,324) of the total catch, followed by *Cx. pipiens* (*s.l*.)/*Cx. torrentium* with 30.86% (*n* = 9,178) and *Ae. vexans* with 18.73% (*n* = 5,571).

**Table 1:** Number of mosquitoes collected in eastern Austria in 2014 and 2015

Taxon (Abbreviation)	Burgenland		Lower Austria		Vienna		Total
	2014	2015	2014	2015	2014	2015	2014/2015
*Coquillettidia richiardii* (*Cq.ric*)	39	4,182	386	3,792	862	63	9,324
*Culex pipiens* (*s.l*.)/*Culex torrentium* (*Cx.pip*)	497	1,829	690	1,590	917	3,655	9,178
*Aedes vexans* (*Ae.vex*)	72	235	4,129	911	216	8	5,571
*Ochlerotatus sticticus* (*Oc.sti*)	68	237	1,045	258	25	4	1,637
*Culex martinii* (*Cx.mar*)	57	986	6	10	3	0	1,062
*Anopheles plumbeus* (*An.plu*)	7	114	111	77	33	5	347
*Aedes cinereus*/*geminus* (*Ae.cin*)	1	24	297	9	1	0	332
*Anopheles hyrcanus* (*An.hyr*)	0	238	1	1	0	1	241
*Culex modestus* (*Cx.mod*)	0	34	2	27	0	0	63
*Anopheles maculipennis* (*s.l*.) (*An.mac*)	6	20	10	20	2	1	59
*Ochlerotatus caspius* (*Oc.cas*)	0	0	17	2	7	2	28
*Ochlerotatus geniculatus* (*Oc.gen*)	2	1	18	4	0	1	26
*Ochlerotatus intrudens* (*Oc.int*)	0	0	14	0	10	0	24
*Ochlerotatus communis* (*Oc.com*)	0	0	8	0	14	0	22
*Anopheles claviger* (*An.cla*)	0	2	0	14	0	0	16
*Ochlerotatus cataphylla* (*Oc.cat*)	0	0	4	5	3	0	12
*Ochlerotatus japonicus* (*Oc. jap*)	5	6	0	0	0	0	11
*Culiseta annulata* (*Cs.ann*)	1	6	0	1	0	2	10
*Uranotaenia unguiculata* (*Ur.ung*)	0	10	0	0	0	0	10
*Ochlerotatus rusticus* (*Oc.rus*)	0	0	4	0	0	0	4
*Anopheles algeriensis* (*An.alg*)	0	3	0	0	0	0	3
*Culex territans* (*Cx.ter*)	0	0	1	0	1	0	2
*Ochlerotatus cantans* (*Oc.can*)	0	0	0	1	1	0	2
*Ochlerotatus flavescens* (*Oc.fla*)	0	0	1	0	0	0	1
*Ochlerotatus leucomelas* (*Oc.leu*)	0	0	0	1	0	0	1
*Aedes*/ *Ochlerotatus* sp.	37	67	670	145	28	5	952
*Anopheles* sp.	1	362	21	25	0	1	410
*Culex* sp.	62	106	29	91	40	59	387
Total	855	8,462	7,464	6,984	2,163	3,807	29,734

In 2014, 20 mosquito species belonging to six genera were detected: *Aedes vexans* represented the most abundant mosquito species with 42.14% (*n* = 4,417), followed by members of the *Cx. pipiens* (*s.l*.)/*Cx. torrentium* with 20.07% (*n* = 2,104) and *Cq. richiardii* with 12.28% (*n* = 1,287). Species collected only in 2014 comprise *Cx. territans*, *Ochlerotatus communis*, *Oc. flavescens*, *Oc. intrudens* and *Oc. rusticus*. In the sampling period in 2015, 21 species belonging to seven genera were detected, with *Cq. richiardii* as the most abundant species comprising 41.74% (*n* = 8,037) of the total catch, followed by *Cx. pipiens* (*s.l*.)/ *Cx. torrentium* (36.74%, *n* = 7,074) and *Ae. vexans* (5.99%, *n* = 1,154). Species collected only in 2015 comprise *An. claviger*, *An. algeriensis*, *Oc. cantans* and *Uranotaenia unguiculata* (Table 1).

### Variation of mosquito abundances between years

Proportional abundances of mosquito species differed significantly between the sampling years (*G*_14_ = 26.153, *P* < 0.05). A significant increase in the proportional abundances of *Cq. richiardii* (*G*_1_ = 109.7, *P* < 0.05), *Cx. pipiens* (*s.l*.)/*Cx. torrentium* (*G*_1_ = 4.758, *P* < 0.05) and *Cx. martinii* (*G*_1_ = 6.5081, *P* < 0.05) was observed from one sampling year to another in eastern Austria over all sampling localities. Proportional abundances of *Ae. vexans* were in contrast found to significantly decrease from 2014 to 2015 (*G*_1_ = 9.1964, *P* < 0.05). *Ochlerotatus sticticus* and *An. plumbeus* follow the same trend over the two years, but likelihood ratio tests did not reveal significant differences. In detail, a significant increase in the proportional abundance of *Cq. richiardii* (*G*_1_ = 106.37, *P* < 0.05) and *Cx. pipiens* (*s.l*.)/*Cx. torrentium* (*G*_1_ = 19.564, *P* < 0.05) in Burgenland province was detected. In Lower Austria province, the proportional abundance of *Cq. richiardii* increased significantly (*G*_1_ = 7.6129, *P* < 0.05), while those of *Ae. vexans* (*G*_1_ = 17.798, *P* < 0.05) decreased significantly between the years. In Vienna province, no significant differences were detected.

#### Spatiotemporal variation in mosquito communities

**Fig. 2:**
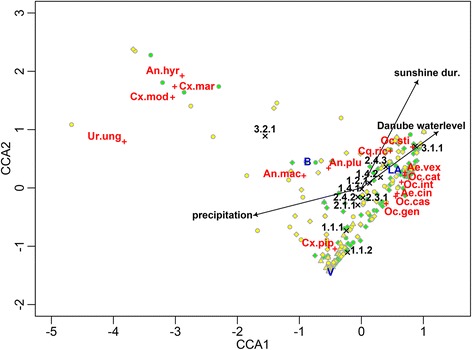
CCA triplot relating mosquito community variation *via* single species responses to environmental parameters. Canonical correspondence analysis identified CORINE land cover types, precipitation, sunshine duration and average maximum Danube water levels as factors (*depicted in black*) affecting abundance patterns of most abundant mosquito species (*depicted in red*, abbreviated as in Table 1). Sites are depicted as circles (Burgenland province), triangles (Lower Austria province) and diamonds (Vienna province), differentiated between 2014 (*green fill*) and 2015 (*yellow fill*); centroids of sites classified into Burgenland (‘B’), Lower Austria (‘LA’) or Vienna (‘V’) province are depicted in blue

Canonical correspondence analysis of communities comprising the most abundant mosquito species in relation to environmental parameters recovered 14 canonical axes, of which the first four significantly explained variation in the dataset (Table 2). The most parsimonious model includes CLC level 3 land cover classes, 14-day-mean precipitation, 14-day-mean sunshine duration and 14-day-mean maximum water level of the Danube River (Fig. 2). In total, the first two axes explain 67%, and the first four axes explain 96% of the variation observed in the dataset (Table 2).

**Table 2:** Canonical correspondence biplot scores for evaluating possible effects of environmental parameters 14 days prior to the sampling date on mosquito species distribution and development

Environmental variable	CCA1	CCA2	CCA3	CCA4
CLC3 3.1.1. Broad-leaved forests	0.48403	0.39831	-0.1162164	-0.50922
CLC3 2.4.2. Complex cultivation patterns	-0.0316	-0.08703	0.0210876	0.019194
CLC3 1.1.1. Continuous urban fabric	-0.13467	-0.40679	0.0544209	0.101226
CLC3 1.1.2. Discontinuous urban fabric	-0.12401	-0.6142	-0.0007399	0.180108
CLC3 1.4.1. Green urban areas	0.00268	-0.0109	0.0519469	-0.004758
CLC3 1.2.1. Industrial or commercial units	0.07393	0.04481	0.46609	-0.212319
CLC3 2.4.3. Land principally occupied by agriculture with significant areas of natural vegetation	0.2204	0.20159	-0.0598521	0.447815
CLC3 3.2.1. Natural grassland	-0.86253	0.49381	-0.1373999	-0.005677
CLC3 2.1.1. Non-irrigated arable land	-0.03196	-0.16288	-0.0329216	0.177909
CLC3 2.3.1. Pastures	0.01392	-0.09426	0.1195053	-0.05643
CLC3 1.4.2. Sport and leisure facilities	0.17149	0.09974	-0.3000622	0.275365
Amount of precipitation	-0.34255	-0.09271	0.6092371	-0.293021
Sunshine duration	0.18	0.36399	0.5682719	0.616774
Water level of the Danube	0.2431	0.18951	-0.1507109	-0.008783

The first CCA axis describes a gradient predominated by increasing Broad-leaved forest (CLC3 3.1.1), Land principally occupied by agriculture with significant areas of natural vegetation (CLC3 2.4.3), Sport and leisure facilities (CLC3 1.4.2), sunshine duration and mean maximum Danube water levels, and decreasing Natural grassland (CLC3 3.2.1), Continuous urban fabric (CLC3 1.1.1), Discontinuous urban fabric (CLC3 1.1.2) and precipitation (Fig. 2, Table 2). The second CCA axis describes a gradient predominated by increasing Broad-leaved forest (CLC3 3.1.1), Land principally occupied by agriculture with significant areas of natural vegetation (CLC3 2.4.3), Natural grassland (CLC3 3.2.1), sunshine duration and mean maximum Danube water level, and decreasing Continuous urban fabric (CLC3 1.1.1) and Discontinuous urban fabric (CLC3 1.1.2) (Fig. 2, Table 2). The gradient described by the third CCA axis is dominated by slightly increasing Industrial or commercial units (CLC3 1.2.1), Pastures (CLC3 2.3.1), sunshine duration and precipitation, and decreasing Broad-leaved forest (CLC3 3.1.1), Natural grassland (CLC3 3.2.1), Sport and leisure facilities (CLC3 1.4.2) as well as decreasing mean maximum Danube water levels (Additional files 1–5: Figures S1–S5); Table 2). The fourth CCA axis describes a gradient of increasing Continuous urban fabric (CLC3 1.1.1), Discontinuous urban fabric (CLC3 1.1.2), Land principally occupied by agriculture with significant areas of natural vegetation (CLC3 2.4.3), Non-irrigated arable land (CLC3 2.1.1), Sport and leisure facilities (CLC3 1.4.2), and strongly increasing sunshine duration, and decreasing Broad-leaved forest (CLC3 3.1.1), Industrial or commercial units (CLC3 1.2.1) and precipitation (Additional files 1–5: Figures: S1–S5); Table 2).

With respect to abundances of single species, the mosquito community was found to be structured along gradients of differentially increasing/decreasing land cover types, climate and hydrological parameters. *Culex martinii*, *An. hyrcanus*, *Cx. modestus* and *Ur. unguiculata* were recovered far from the ordination centre and were found to be associated with Natural grassland (CLC3 3.2.1), relatively high sunshine duration, and high precipitation (Fig. 2, Tables 2 and 3).

**Table 3:** Environmental parameters tested using PERMANOVA based on distance matrices (adonis():’*vegan*’ package (Oksanen et al. [54])

Environmental parameters	*df*	Sums of squares	Mean squares	*F*	*R* ^2^	Pr(>F)
Province	2	5.954	297.717	147.173	0.09416	0.001***
CLC3	11	13.981	127.101	62.831	0.22109	0.001***
Danube water level (14 days)	1	0.643	0.64250	31.761	0.01016	0.001***
Air temperature (14 days)	1	0.579	0.57925	28.635	0.00916	0.052
Relative air humidity (14 days)	1	0.541	0.54142	26.765	0.00856	0.035*
Sunshine duration (14 days)	1	0.693	0.69263	34.239	0.01095	0.002**
Amount of precipitation (14 days)	1	0.241	0.24102	11.915	0.00381	0.313
Distance to the nearest wetland	1	0.437	0.43724	21.615	0.00691	0.001***
CLC3: Distance to the nearest wetland	3	1.731	0.57697	28.522	0.02737	0.026*
Residuals	190	38.435	0.20229		0.60781	
Total	212	63.236			100.000	

Asterisks indicate significant effects of certain environmental parameters (*, *P*< 0.05; **, *P *< 0.01; ***, *P* < 0.001)

*Anopheles maculipennis* (*s.l*.) and *An. plumbeus* were recovered close to one another, and relatively close to the ordination origin. These species were found to be associated with Natural grassland (CLC3 3.2.1), Land principally occupied by agriculture with significant areas of natural vegetation (CLC3 2.4.3), average Danube water levels, and slightly higher precipitation (Fig. 2, Tables 2 and 3). *Anopheles hyrcanus*, *An. maculipennis* (*s.l*.), *An. plumbeus*, *Cx. martinii*, *Cx. modestus* were predominately collected in Burgenland and *Ur. unguiculata* was exclusively collected in this province (Fig. 2, Tables 2 and 3). *Culex pipiens* (*s.l*.)/*Cx. torrentium* was associated with continuous urban fabric (CLC3 1.1.1), Discontinuous urban fabric (CLC3 1.1.2), Non-irrigated arable land (CLC3 2.1.1), high precipitation, relatively low sunshine and below average mean maximum Danube water level. Furthermore, this species group was predominately collected in Vienna (Fig. 2, Table 2). *Coquillettidia richiardii* was positively associated with Land principally occupied by agriculture with significant areas of natural vegetation (CLC3 2.4.3), Broad-leaved forest (CLC3 3.1.1), high sunshine duration and slightly elevated Danube water levels, and was negatively associated with precipitation (Fig. 2, Table 2). *Ochlerotatus sticticus* was strongly related to Broad-leaved forest (CLC3 3.1.1), and Land principally occupied by agriculture with significant areas of natural vegetation (CLC3 2.4.3), elevated Danube water levels, and above average sunshine duration (Fig. 2, Tables 2 and 3). The taxa *Ae. vexans*, *Ae. cinereus*/*geminus*, *Oc. cataphylla*, *Oc. intrudens*, *Oc. caspius* and *Oc. geniculatus* were associated with Broad-leaved forest (CLC3 3.1.1), Land principally occupied by agriculture with significant areas of natural vegetation (CLC3 2.4.3), Sport and leisure facilities (CLC3 1.4.2), high sunshine duration, high Danube water levels and low precipitation (Fig. 2, Tables 2 and 3). *Aedes vexans* was more strongly related to Broad-leaved forest (CLC3 3.1.1) than the other taxa. Furthermore, these species and *Cq. richiardii* and *Oc. sticticus* were mostly collected in Lower Austria (Fig. 2).

PERMANOVA indicated significant effects of environmental parameters on mosquito community composition. Communities differed significantly between provinces and CLC3 land cover types (Table 3).

**Fig. 3:**
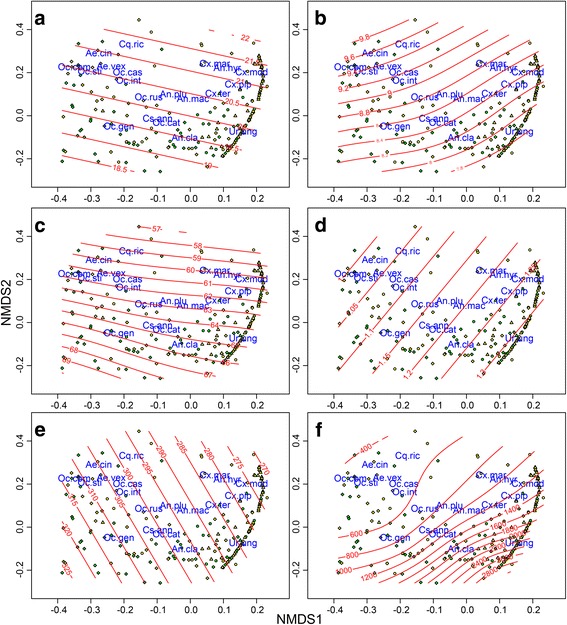
NMDS representation of Bray-Curtis dissimilarities between unique sampling events in eastern Austria. Mosquito communities are clearly differentiated from one another, and in relation to environmental parameters depicted as red isolines modelled in the ordination space defined by the first two NMDS axes, using a general additive modelling approach. Communities dominated by certain species exhibit differentiation in ordination space according to species’ environmental niches. **a** Temperature variability. **b** Sunshine duration variability. **c** Relative humidity variability. **d** Precipitation variability. **e** Average maximum Danube water level variability. **f** Distances of communities to the nearest wetland. Air temperature and precipitation patterns were not found to structure communities. Weighted average species scores are provided in blue; sites are depicted as circles (Burgenland), triangles (Lower Austria) and diamonds (Vienna), differentiated between 2014 (*green fill*) and 2015 (*yellow fill*). 3D stress 0.97

Community composition was also significantly influenced by mean maximum Danube water levels, relative air humidity, sunshine duration, the distance to the nearest wetland, and the interaction term between CLC3 land cover types and distance to the nearest wetland. In addition, there seemed to be an effect of air temperature, but this was not significant (Table 3). Generalised additive modelling of environmental parameters in ordination space suggested communities characterised by high abundances of *Ae. vexans*, *Ae. cinereus*/*geminus*, *Oc. sticticus*, *Oc. communis*, *Oc. intrudens*, *Oc. caspius* and *Cq. richiardii* to be located close to wetlands (Fig. 3). The same communities were also associated with high sunshine duration and high mean maximum Danube water levels. Communities were structured along a sunshine duration gradient. Also, the distance of sampling site to the nearest wetland (Fig. 3) and the interaction term of CLC3 land cover types and distance to the nearest wetland significantly influence community composition. While PERMANOVA did not identify significant effects of precipitation, mosquito communities dominated by certain taxa seemed to be associated with slightly higher precipitation (Fig. 3).

## Discussion

### Species inventory and medical importance of the mosquito community in eastern Austria

In Austria 46 species belonging to seven genera are known to exist at least sporadically. The last valid species list for Austria was published in 2002 by the Federal Ministry of Agriculture, Forestry, Environment and Water Management in Austria as part of the Fauna Aquatica Austriaca [60] comprising 39 species belonging to six genera (including the valid genus *Ochlerotatus* as a subgenus of genus *Aedes*). Since 2011 seven more species have been reported in Austria: *Oc. nigrinus*, *Ae. albopictus*, *An. hyrcanus*, *Oc. japonicus japonicus*, *Culiseta longiareolata*, *Oc. riparius* and *Oc. cyprius* [61–67]. In this study, 21 of the 46 species known from Austria were re-confirmed for eastern Austria. Moreover, our sampling included eight species that are not differentiable based on female semaphoronts, but are known to or potentially occur in Austria. Our sampling effort thus represents 45–63% of the whole Austrian species inventory, including several rare taxa and we assume that the mosquito community characterized here is representative, and comprises the majority of taxa to be expected in eastern Austria.

Only a few medically important mosquito taxa were found in high abundances (*Cx. pipiens* (*s.l*.), *Cq. richiardii* and *Ae. vexans*). While this indicates the presence of a large latent pool of vectors for medically important pathogens such as WNV, avian malaria and filarioid helminths, potent vectors of human pathogens (like *P. vivax*) are not present in high densities. We thus assume a low potential for the establishment and propagation of human mosquito-borne pathogens in eastern Austria under the prevailing environmental conditions. However, as presence and abundance of mosquito taxa greatly depend on the environment, the on-going global change could alter mosquito community composition and proportional abundances.

### Spatiotemporal variation of mosquito communities and single species abundance

We found mosquito communities and abundances of single species to be mostly affected by land cover types. Our analyses indicate a distinct niche differentiation of mosquito species in eastern Austria. We found high numbers of most abundant taxa to be largely influenced by gradients of habitat types, with climatic parameters mostly controlling temporal variation. In particular, the observed shifts in species abundance and occurrence patterns between the sampling years can be related to differences in meteorological parameters. While 2014 was dominated by so-called floodplain mosquitoes, species depending on sufficient precipitation to replenish potential larval habitats were more abundant in 2015. Nevertheless, the impact of land cover type on community composition and abundance was evident.

Abundances of floodplain related species like *Ae. vexans*, *Oc. sticticus* or *Oc. caspius* were positively related to the mean maximum Danube water level, but not to the amount of precipitation, indicating hydroperiod as a substantial variable for the providence of breeding habitats. Moreover, these species were found to be closely associated with Broad-leaved forest (CLC3 3.1.1), but this relationship might not be causal: most of the sampling sites in this land cover type were located in floodplains. However, as floodplains feature typical vegetation, it is likely that this land cover type is an informative predictor for these species if integrated with information on waterways. A strong relationship between annual mosquito abundance and hydroperiod was also observed in Mediterranean wetlands, where most of the *Culex* species investigated were positively related to hydroperiod [22]. The observed positive effect of sunshine duration on *Ae. vexans* and *Oc. sticticus* can be related to their phenology, as they mostly occur in summer.

We found *Cq. richiardii* related to Broad-leaved forest (CLC3 3.1.1) and Land principally occupied by agriculture with significant areas of natural vegetation (CLC 2.4.3) which both provide permanent water bodies with suitable host plants such as *Typha* sp., *Juncus* sp. and *Phragmites* sp. [12]. In particular, densely vegetated irrigation channels and backwaters are highly abundant in these land cover types. These habitat types also provide water quality and water movement parameters as required by the genus [68]. The positive relation to sunshine duration furthermore characterises this taxon as a summer species.

A single taxon, *Cx. pipiens* (*s.l*.)/*Cx. torrentium*, was found to be closely associated with urban habitats (CLC3 1.1.1 and CLC3 1.1.2). While this appears to be in contrast to findings of our earlier study on a dataset comprising only samples taken in 2014 (where these were found mostly in agriculturally dominated land cover types; [49]), it rather demonstrates the euryoecious character of the taxa. Indeed, in a year with lower-than-average precipitation in the study area, *Cx. pipiens* (*s.l*.)/*Cx. torrentium* mostly colonise habitats in agricultural areas in which aquatic habitats in the form of irrigation channels or semi-permanent puddles of irrigation water are present, but also urbanised areas in which anthropogenic standing waters (e.g. rain water barrels) are present. Once average precipitation patterns prevail, the taxa colonise every available larval habitat, including water filled anthropogenic crevices, copiously available in urban areas. Indeed, the *Cx. pipiens* complex is described to be ubiquitous with breeding sites in urban, natural, and rural/agricultural environments [8], confirming this hypothesis. Additionally, a similar relationship between this taxon and precipitation was observed in the Po Plain [69]. This also might be the reason why *Cx. pipiens* complex taxa are repeatedly described as preferring urban habitats cf. [70, 71] - their euryoecious nature potentially results in colonisation of all available larval habitats, including those in urban areas. The negative relationship of the taxon and sunshine duration relates to the seasonality of the group, as the highest abundances occur in late summer to fall.

Interestingly, we found abundances of other *Culex* taxa, like *Cx. modestus*, related to Natural grassland (CLC3 3.2.1), but also to precipitation. Thus this taxon potentially relies on sufficient precipitation to create adequate larval habitats in these landscapes. The relative position of *Cx. martinii* in the CCA-plot indicates that this species might prefer the same habitat type, and might as well be dependent on precipitation to generate suitable larval habitats. Intriguingly, *Culex* taxa (*Cx. pipiens* (*s.l*.), *Cx. torrentium*) capable of transmitting WNV [72] and *Dirofilaria immitis* and *D. repens* [73], and *Cx. modestus*, which have been demonstrated to potentially transmit Tahyna virus [11], favour completely different habitats in eastern Austria. This indicates that mosquito monitoring schemes, aiming to address the spread of potential vector populations and infectious disease, need to be adequately designed.

Mosquito community composition differed significantly between land cover types and provinces. Further, climatic parameters (relative air humidity, sunshine duration) and geography (distance to the nearest wetland, and the interaction term between CLC3 land cover types and distance to the nearest wetland) structured mosquito communities. This indicates that abundance and occurrence of certain taxa are strictly linked to the availability of habitat or climatic niches. For example, the Mediterranean species *Ur. unguiculata* and *An. algeriensis* were found only in Burgenland province, dominated by Pannonian climate. Interestingly, some species adapted to hydroperiod variation, like some *Ochlerotatus* spp. (e.g. *Oc. sticticus*, *Oc. communis* and *Oc. intrudens*; [42]) or *Aedes* spp. (e.g. *Ae. vexans* and *Ae. cinereus*) were mostly found in 2014 in Lower Austria, the province comprising the largest floodplain areas. Furthermore, communities dominated by these species are geographically close to wetlands and are characterised by experiencing high mean maximum Danube water levels 14 days prior to sampling. However, communities dominated by mosquito taxa tending to use permanent water bodies or rainwater-dependent breeding sites are depicted at a larger distance to wetlands. Also, these communities seem to be dependent on higher levels of relative humidity, and slightly elevated precipitation, potentially linked to larval habitat stability. In addition, communities were structured along a temperature gradient, related to their seasonality: species emerging early or late in the year dominated communities that were depicted at relatively lower air temperatures. Similarly, the same communities were associated with relatively lower sunshine duration, indicative of their phenology. Both air temperature and sunshine duration structured mosquito species along an environmental gradient, and clearly separated spring and autumn species from summer species, with multivoltine species occurring throughout the sampling period: occurring earliest in late summer, *Ur. ungiuculata* was most abundant when daily sunshine duration was low; the summer species *Cq. richiardii*, *Ae. cinereus*/*geminus*, *Oc. communis*, *Oc. sticticus*, *Ae. vexans*, *Oc. intrudens* and *Oc. caspius* were most abundant when sunshine duration was high; mulitvoltine *Culex* and *Anopheles* mosquitoes occurred throughout the sampling season from April to October.

Interestingly, we seemingly found an ecological differentiation at genus level: *Aedes* and *Ochlerotatus* species are more abundant at sampling events with higher water levels, while *Culex* and *Anopheles* species mostly occur at sampling events with lower water levels.

### Habitat preferences and climatic niche of non-indigenous species

Invasive alien species are taxa that are introduced outside their native range, the introduction and spread of which has an impact on indigenous species and biological diversity [74]. The dispersal of invasive mosquito species is driven by human transcontinental mobility and international trade, whereas their establishment is facilitated or prevented by climate conditions [75]. The presence of a suitable climatic niche is a key factor for the establishment of a species in a new area [75] demonstrated by *Oc. j. japonicus*, adapted to temperate climates [76], in parts of eastern Austria [77]. However, surveys in Europe in areas where this species is already established indicate a tendency of this species to favour forest habitats, but to prefer alternative environments for oviposition due to a lack of suitable breeding habitats, biasing their habitat preference [78]. Also, Bartlett-Healy et al. [79] observed *Oc. j. japonicus* larvae more frequently in wooden/rural areas than in urban sites. Furthermore, this species is adapted to temperate climates with the capacity/ability to withstand winters such as occur in its native range in northern Japan [76]. Accordingly, *Oc. j. japonicus* was reported to be more abundant in container breeding habitats with lower mean temperatures [79]. *Anopheles hyrcanus* and *Cs. longiareolata*, both widely distributed species in the Mediterranean, were likely not introduced by global trade, but rather traced their climatic niche northwards as climatic conditions became more favourable. Nevertheless, *An. hyrcanus* was only found in specific habitat types: natural grassland areas near Lake Neusiedl with partly temporary and perennially water bodies ([64], this study), and in the Danube floodplain near sun-exposed stagnant water bodies rich in reeds [65]. In contrast, *Cs. longiareolata* is reported to prefer artificial water bodies in Austria [64, 66], instead of natural ones, contradicting literature (e.g. [11]). However, to assess the current and potential future distribution of both species, larval breeding habitats need to be more intensively surveyed. Abundance fluctuations observed in *An. hyrcanus*, where abundance increased rapidly from one year to another in Burgenland (Table 1), indicate the necessity of more intense sampling of aquatic habitats. Such larval surveys are crucial to estimating if a non-native species finds and colonises larval habitats, and if and how this might impact native Culicidae species in this area.

### Relevance of habitat characteristics on mosquito communities and species abundance

Our study clearly demonstrates the importance of habitat structure on mosquito community composition and abundance of single species. Thus, we confirm results of earlier studies indicating significant effects of habitat types on mosquito species [21–23]. Furthermore, we detected effects based on the most detailed CORINE land cover classification level, indicating that utilisation of higher-level classification might unjustifiably disregard available information. Mosquito communities evidently are structured by land cover type and are furthermore affected by their relative distance to the nearest wetland and climatic conditions. This underlines the adaption of mosquito species to different water resources and hydroperiod dynamics. While development of some species was found to be favoured by or depend on increasing water levels, others can be negatively affected [22]. The environmental niches used, especially those defined by the land cover type and distances to the wetland areas, not only affect mosquito communities but also populations of potential hosts. Near natural or natural areas will be inhabited by completely different host communities compared to strictly urban areas.

In turn, this results in a causality dilemma concerning the identification of factors structuring mosquito communities. Whether mosquitoes choose a suitable habitat and feed on the most abundant host or aggregate around their preferred host which is adapted to a specific environment [19] remains yet unknown. The recent findings of strongly overlapping host feeding patterns in different mosquito species [25] contradicting the usually described host preferences (e.g. [9, 80]) underline the necessity of combining habitat structure and availability as well as host populations and host feeding patterns in future studies on mosquito communities. Furthermore, for a higher resolution of relationships between habitat structure and mosquito distribution data, larval surveys should be included in future studies, since adult surveys tend to underestimate species abundance in some cases (cf. [9]).

## Conclusions

While the CORINE land cover database is commonly used to classify habitats for subsequent comparison of species abundance or mosquito community composition, it is common to use higher-level CLC classifications [25, 81]. In this contribution, we show that habitat structure has a high impact on spatial variation of mosquito community composition and species abundances, while climatic parameters mostly affect temporal abundance patterns in mosquito species. Our results confirm our a priori hypotheses concerning differentiation of mosquito species along land cover/climate gradients and furthermore demonstrate distinct effects of meteorological seasonality on mosquito phenology. This indicates that habitat structure is a significant driver of mosquito diversity, and needs to be included when aiming to predict future mosquito occurrence and abundance scenarios, and potential invasions of allochthonous species. In particular, resolution of models aiming to estimate potential propagation scenarios for medically important mosquito species and mosquito-borne pathogens will greatly benefit from this source of information.

## Additional files

Additional file 1: Figure S1. CCA triplot relating mosquito community variation *via* single species responses to environmental parameters based on significant CCA axes 1 and 3. Canonical correspondence analysis identified CORINE land cover types, precipitation, sunshine duration and average maximum Danube water levels as factors (*depicted in black*) affecting abundance patterns of most abundant mosquito species (*depicted in red*, abbreviated as in Table 1). Sites are depicted as circles (Burgenland province), triangles (Lower Austria province) and diamonds (Vienna province), differentiated between 2014 (*green fill*) and 2015 (*yellow fill*); centroids of sites classified into Burgenland (‘B’), Lower Austria (‘LA’) or Vienna (‘V’) province are depicted in blue. (TIF 464 kb)

Additional file 2: Figure S2. CCA triplot relating mosquito community variation *via* single species responses to environmental parameters based on significant CCA axes 1 and 4. Canonical correspondence analysis identified CORINE land cover types, precipitation, sunshine duration and average maximum Danube water levels as factors (*depicted in black*) affecting abundance patterns of most abundant mosquito species (*depicted in red*, abbreviated as in Table 1). Sites are depicted as circles (Burgenland province), triangles (Lower Austria province) and diamonds (Vienna province), differentiated between 2014 (*green fill*) and 2015 (*yellow fill*); centroids of sites classified into Burgenland (‘B’), Lower Austria (‘LA’) or Vienna (‘V’) province are depicted in blue. (TIF 471 kb)

Additional file 3: Figure S3. CCA triplot relating mosquito community variation *via* single species responses to environmental parameters based on significant CCA axes 2 and 3. Canonical correspondence analysis identified CORINE land cover types, precipitation, sunshine duration and average maximum Danube water levels as factors (*depicted in black*) affecting abundance patterns of most abundant mosquito species (*depicted in red*, abbreviated as in Table 1). Sites are depicted as circles (Burgenland province), triangles (Lower Austria province) and diamonds (Vienna province), differentiated between 2014 (*green fill*) and 2015 (*yellow fill*); centroids of sites classified into Burgenland (‘B’), Lower Austria (‘LA’) or Vienna (‘V’) province are depicted in blue. (TIF 505 kb)

Additional file 4: Figure S4. CCA triplot relating mosquito community variation *via* single species responses to environmental parameters based on significant CCA axes 2 and 4. Canonical correspondence analysis identified CORINE land cover types, precipitation, sunshine duration and average maximum Danube water levels as factors (*depicted in black*) affecting abundance patterns of most abundant mosquito species (*depicted in red*, abbreviated as in Table 1). Sites are depicted as circles (Burgenland province), triangles (Lower Austria province) and diamonds (Vienna province), differentiated between 2014 (*green fill*) and 2015 (*yellow fill*); centroids of sites classified into Burgenland (‘B’), Lower Austria (‘LA’) or Vienna (‘V’) province are depicted in blue. (TIF 470 kb)

Additional file 5: Figure S5. CCA triplot relating mosquito community variation *via* single species responses to environmental parameters based on significant CCA axes 3 and 4. Canonical correspondence analysis identified CORINE land cover types, precipitation, sunshine duration and average maximum Danube water levels as factors (*depicted in black*) affecting abundance patterns of most abundant mosquito species (*depicted in red*, abbreviated as in Table 1). Sites are depicted as circles (Burgenland province), triangles (Lower Austria province) and diamonds (Vienna province), differentiated between 2014 (*green fill*) and 2015 (*yellow fill*); centroids of sites classified into Burgenland (‘B’), Lower Austria (‘LA’) or Vienna (‘V’) province are depicted in blue. (TIF 477 kb)

## Abbreviations

CLC 3 2.4.2: Complex cultivation patterns
CLC: Corine land cover
CLC3 1.1.1: Continuous urban fabric
CLC3 1.1.2: Discontinuous urban fabric
CLC3 1.2.1: Industrial or commercial units
CLC3 1.4.1: Green urban areas
CLC3 1.4.2: Sport and leisure facilities
CLC3 2.1.1: Non-irrigated arable land
CLC3 2.3.1: Pastures
CLC3 2.4.3: Land principally occupied by agriculture, with significant areas of natural vegetation
CLC3 3.1.1: Broad-leaved forest
CLC3 3.2.1: Natural grassland
CLC3 CORINE: Land cover level 3 (highest level)
CORINE: Co-ordinated information on the environment
