# Supplementary figures and images for: Landscape structure affects distribution of potential disease vectors (Diptera: Culicidae)
Source: Parasit Vectors. 2017 Apr 26;10:205. doi: 10.1186/s13071-017-2140-6 (PMC5405510; doi:10.1186/s13071-017-2140-6)

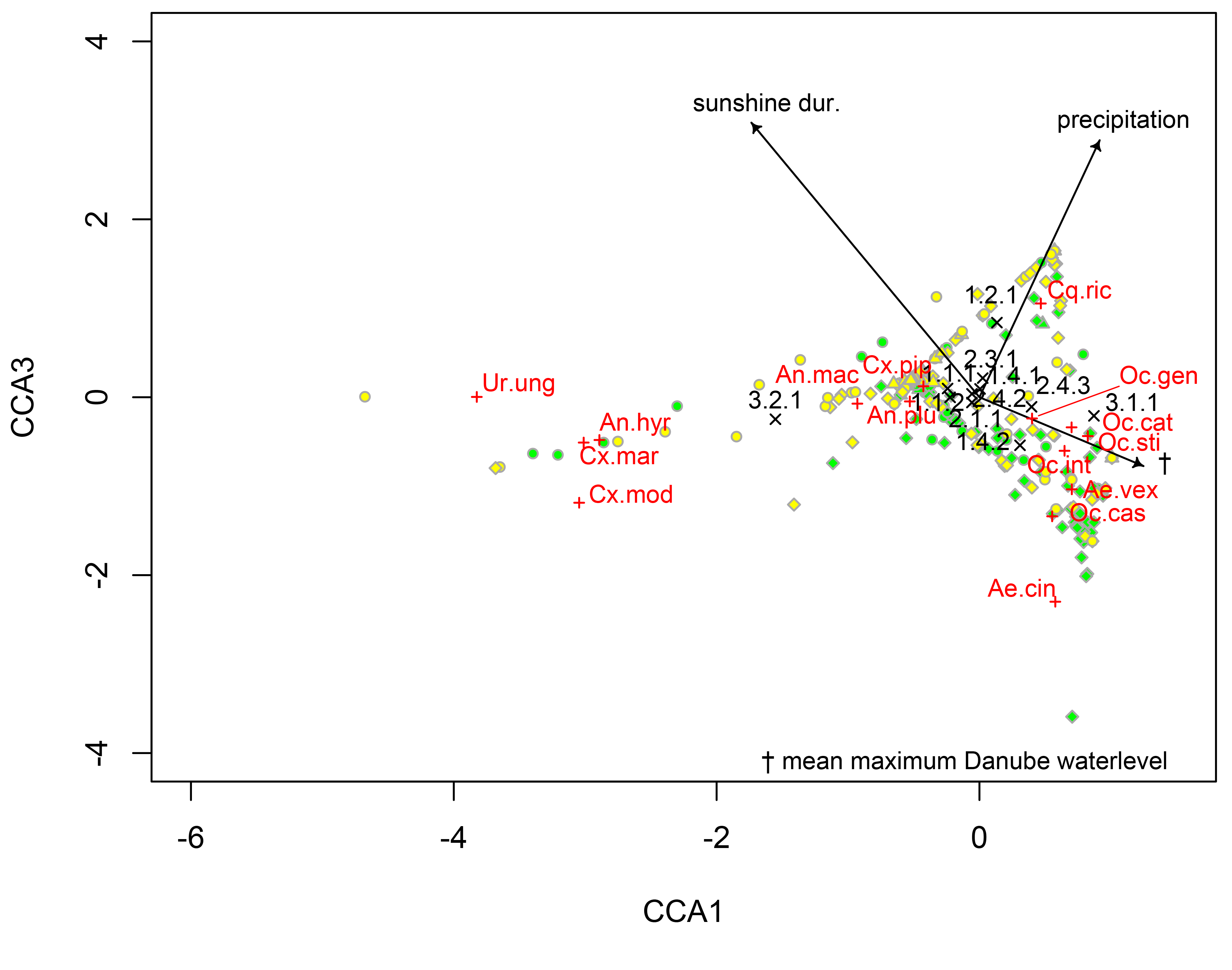

Supplement: Supplementary file 1 — CCA triplot relating mosquito community variation via single species responses to environmental parameters based on significant CCA axes 1 and 3. Canonical correspondence analysis identified CORINE land cover types, precipitation, sunshine duration and average maximum Danube water levels as factors (depicted in black) affecting abundance patterns of most abundant mosquito species (depicted in red, abbreviated as in Table 1). Sites are depicted as circles (Burgenland province), triangles (Lower Austria province) and diamonds (Vienna province), differentiated between 2014 (green fill) and 2015 (yellow fill); centroids of sites classified into Burgenland (‘B’), Lower Austria (‘LA’) or Vienna (‘V’) province are depicted in blue. (TIF 464 kb) [file 13071_2017_2140_MOESM1_ESM.tif]

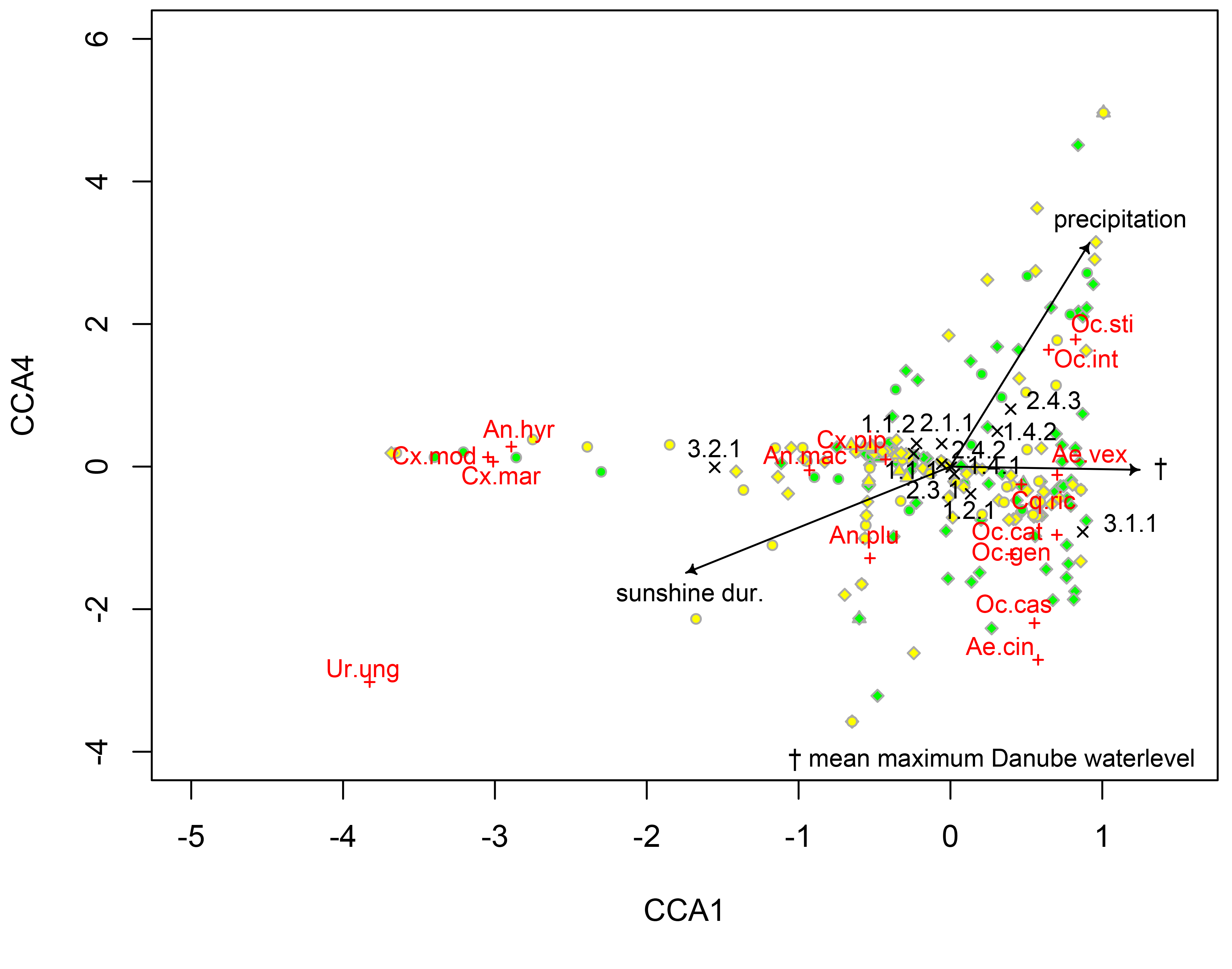

Supplement: Supplementary file 2 — CCA triplot relating mosquito community variation via single species responses to environmental parameters based on significant CCA axes 1 and 4. Canonical correspondence analysis identified CORINE land cover types, precipitation, sunshine duration and average maximum Danube water levels as factors (depicted in black) affecting abundance patterns of most abundant mosquito species (depicted in red, abbreviated as in Table 1). Sites are depicted as circles (Burgenland province), triangles (Lower Austria province) and diamonds (Vienna province), differentiated between 2014 (green fill) and 2015 (yellow fill); centroids of sites classified into Burgenland (‘B’), Lower Austria (‘LA’) or Vienna (‘V’) province are depicted in blue. (TIF 471 kb) [file 13071_2017_2140_MOESM2_ESM.tif]

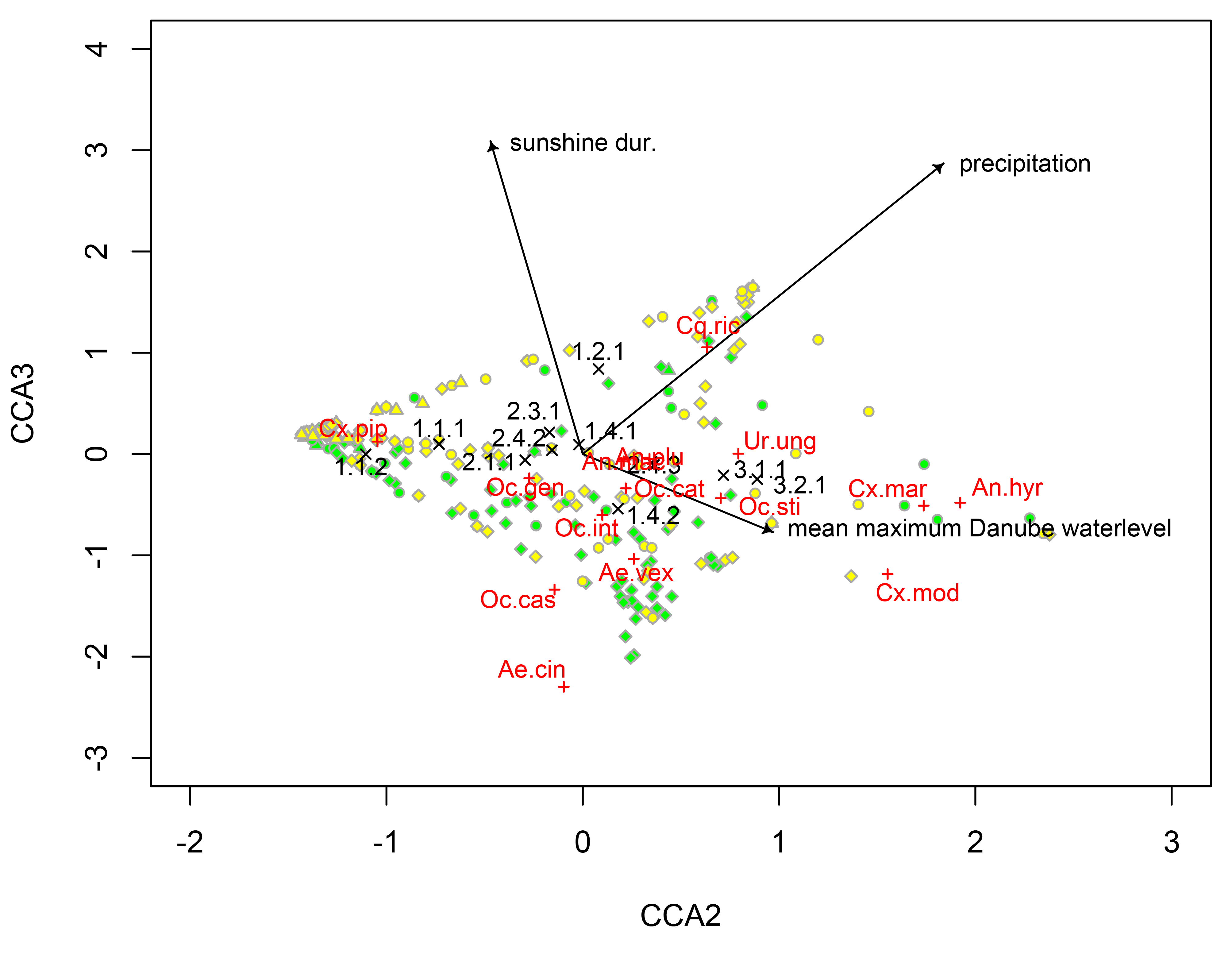

Supplement: Supplementary file 3 — CCA triplot relating mosquito community variation via single species responses to environmental parameters based on significant CCA axes 2 and 3. Canonical correspondence analysis identified CORINE land cover types, precipitation, sunshine duration and average maximum Danube water levels as factors (depicted in black) affecting abundance patterns of most abundant mosquito species (depicted in red, abbreviated as in Table 1). Sites are depicted as circles (Burgenland province), triangles (Lower Austria province) and diamonds (Vienna province), differentiated between 2014 (green fill) and 2015 (yellow fill); centroids of sites classified into Burgenland (‘B’), Lower Austria (‘LA’) or Vienna (‘V’) province are depicted in blue. (TIF 505 kb) [file 13071_2017_2140_MOESM3_ESM.tif]

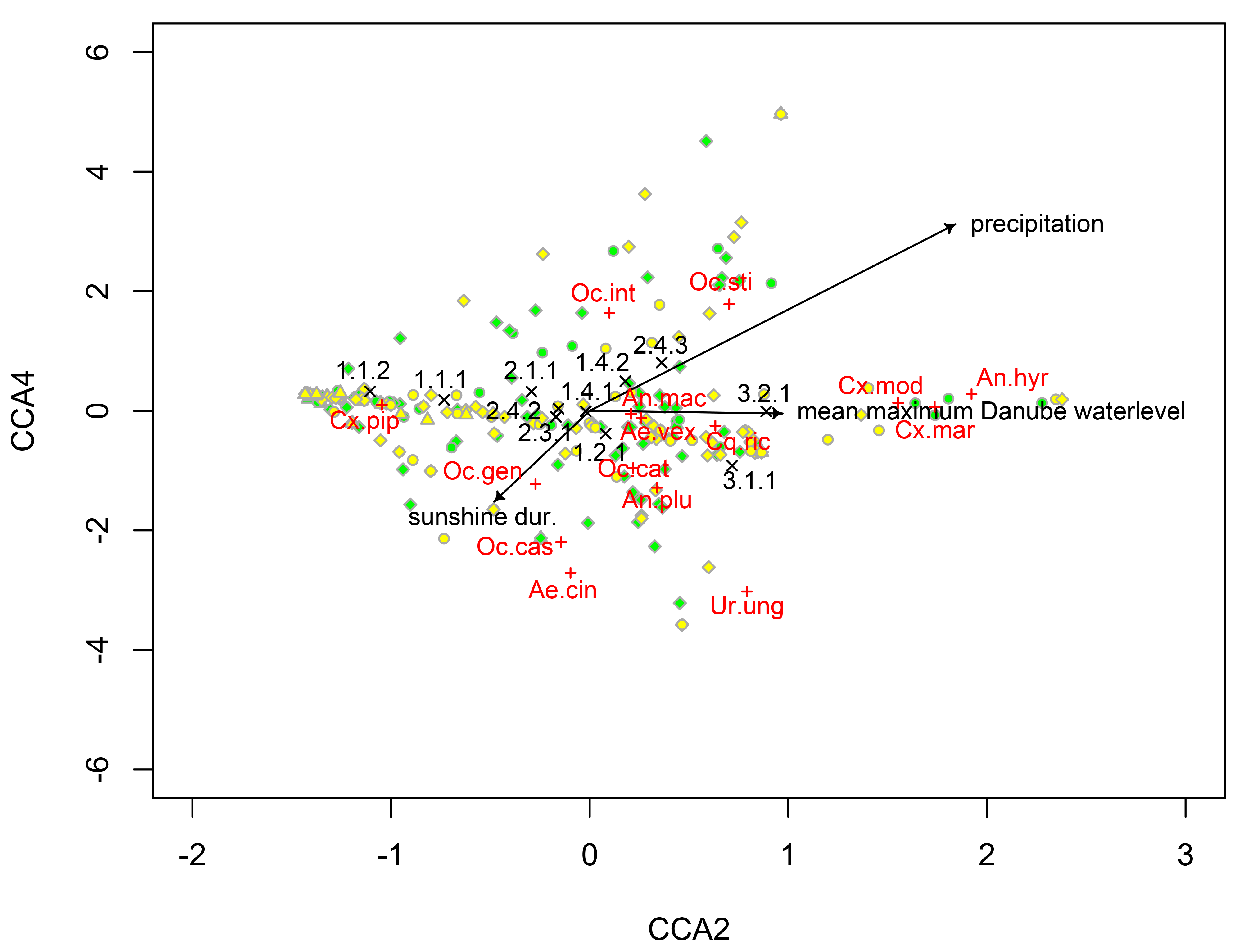

Supplement: Supplementary file 4 — CCA triplot relating mosquito community variation via single species responses to environmental parameters based on significant CCA axes 2 and 4. Canonical correspondence analysis identified CORINE land cover types, precipitation, sunshine duration and average maximum Danube water levels as factors (depicted in black) affecting abundance patterns of most abundant mosquito species (depicted in red, abbreviated as in Table 1). Sites are depicted as circles (Burgenland province), triangles (Lower Austria province) and diamonds (Vienna province), differentiated between 2014 (green fill) and 2015 (yellow fill); centroids of sites classified into Burgenland (‘B’), Lower Austria (‘LA’) or Vienna (‘V’) province are depicted in blue. (TIF 470 kb) [file 13071_2017_2140_MOESM4_ESM.tif]

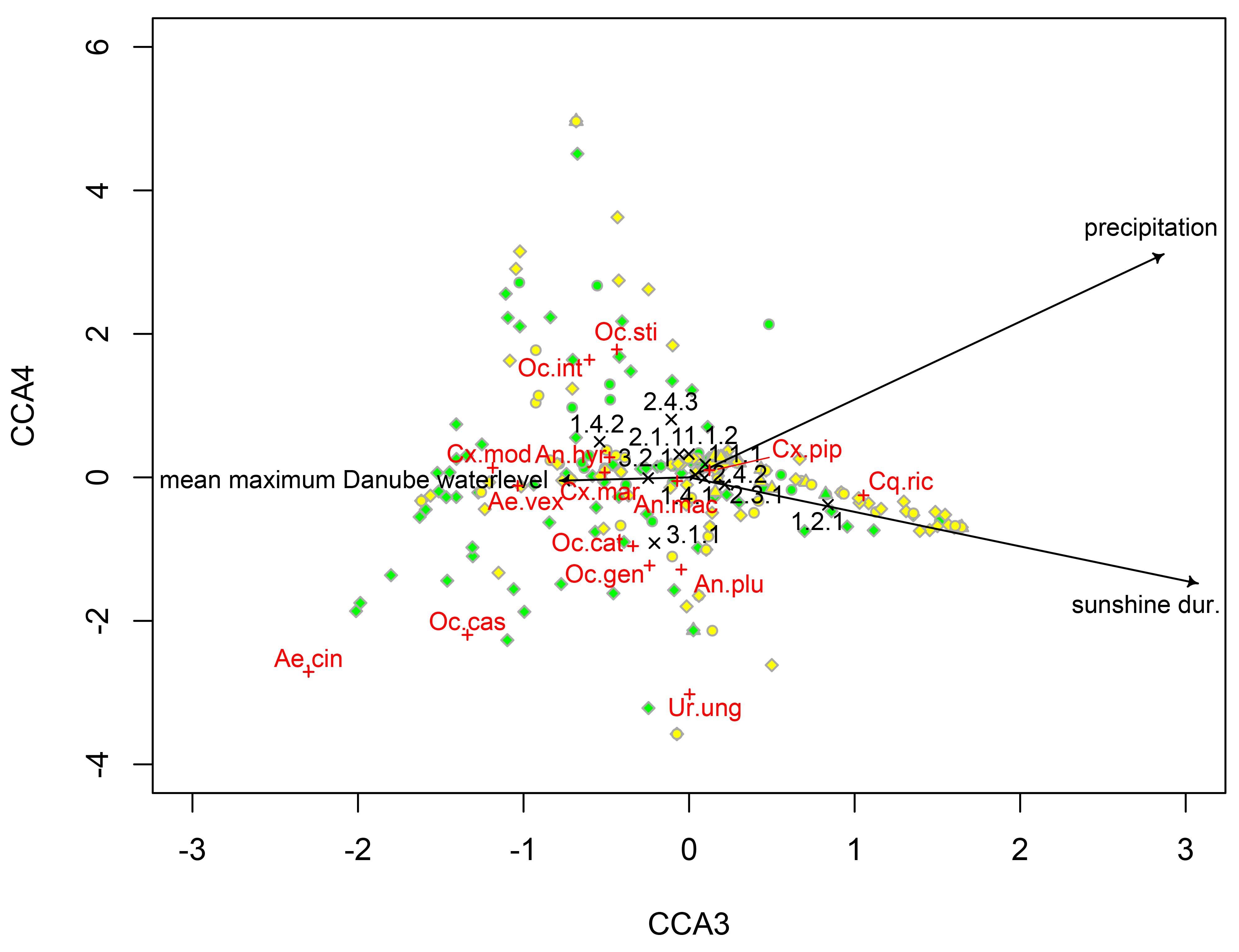

Supplement: Supplementary file 5 — CCA triplot relating mosquito community variation via single species responses to environmental parameters based on significant CCA axes 3 and 4. Canonical correspondence analysis identified CORINE land cover types, precipitation, sunshine duration and average maximum Danube water levels as factors (depicted in black) affecting abundance patterns of most abundant mosquito species (depicted in red, abbreviated as in Table 1). Sites are depicted as circles (Burgenland province), triangles (Lower Austria province) and diamonds (Vienna province), differentiated between 2014 (green fill) and 2015 (yellow fill); centroids of sites classified into Burgenland (‘B’), Lower Austria (‘LA’) or Vienna (‘V’) province are depicted in blue. (TIF 477 kb) [file 13071_2017_2140_MOESM5_ESM.tif]
